# ‘Blue Route’ for combating climate change

**DOI:** 10.1093/nsr/nwab099

**Published:** 2021-05-29

**Authors:** Gang Liu (刘刚), Jiuhui Qu (曲久辉), Mark van Loosdrecht

**Affiliations:** Research Centre for Eco-Environmental Sciences, Chinese Academy of Sciences, China; Research Centre for Eco-Environmental Sciences, Chinese Academy of Sciences, China; Department of Biotechnology, Delft University of Technology, the Netherlands

## Abstract

Today's urban water system can be transformed, without big modifications, into a natural and revolutionary “Blue Route” for combating climate change and constructing carbon neutral city, which will lead to self-sustainability of the water sector and supply energy and resources to other sectors.

The ongoing pandemic has demonstrated to the world the drastic consequences of a strong global economic shock, while there is no doubt that the human and economic consequences of extreme climate events or rising sea levels could far exceed those of the current crisis. It is unfortunate that the 26th UN Climate Change Conference of the Parties (COP26), planned to be held in November 2020 in Glasgow, UK, had to be postponed to next November. Nevertheless, after China promised to become carbon neutral before 2060 and to begin cutting its emissions within the next 10 years in September 2020, hopes of limiting global warming to below 2°C have reignited and the world's attention was again directed to the urgent need to make serious progress to achieve this goal [1]. Though energy and transport sectors have tended to be the focus of attention in this effort, it is essential that all sectors take action to curb climate change [2].

For the water sector, its potential contribution for combating climate change has long been neglected. Traditionally, the water sector has been seen as unavoidably energy-intensive, as it is focused on health and protection, a feature that will only be worsened by global warming, thus further aggravating climate change [3]. The most recent successful initiatives that integrate energy and resource recovery in the water sector show that the traditional energy-intensive view is mistaken. Without building from scratch or making big modifications, today's urban water system can be transformed into a natural and revolutionary ‘Blue Route’ to combat climate change. This Blue Route involves building a greener urban water sector and supplying energy and resources to other sectors (Fig. 1). It would not only lead to self-sustainability of the water sector, but also make a significant contribution to emission reduction across different sectors.

**Figure 1.**
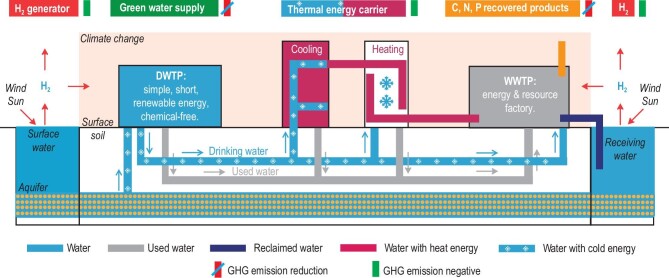
Illustration of the ‘Blue Route’ for combating climate change. (i) Renewable energy for water sector and nature-based, chemical-free water purification, maintaining cold energy while passing through aquifer soil. (ii) Green hydrogen generation from water splitting to meet energy needs of water and other sectors. (iii) Water as thermal energy carrier for cooling and heating, distributed through existing urban water infrastructure. (iv) Energy, and C, N, P, S resource capturing as single-cell protein from wastewater. DWTP, drinking water treatment plant; WWTP, wastewater treatment plant.

The development of greener urban water systems includes using renewable energy, promoting nature-based purification, reducing chemical dosage rates and introducing energy and resource recovery. The drinking water systems can be made even greener through the use of nature-based aquifer purification combined with tight membrane processes; this would stabilize the water temperature for thermal energy recovery, and render the process chemical-free through robust performance on contaminants (especially pathogens) removal, thus completely avoiding the need for the dosing of coagulants and disinfectants [4]. Besides greenhouse gas (GHG) emission reduction, it would help to control the formation of toxic by-products and the deterioration of water infrastructure [5]. Benefiting from the great performance of pretreatment by riverbank filtration, the subsequent one-step reverse osmosis (OSRO) plant for drinking water production (2 × 10^6^ m^3^/year, Oasen, the Netherlands) demonstrated that OSRO driven by wind energy would have lower investment cost (12–14 million EUR), operation cost (0.4 EUR/m^3^) and CO_2_ emission (∼0.3 kg CO_2_eq/m^3^) than that of a traditional drinking water treatment plant [6].

Storage and transportation are bottlenecks to the implementation of renewable energy systems, and present important challenges, for instance, in meeting the demand for electricity during the night or on cloudy days using solar energy. But photoelectrochemical water-splitting cells offer the possibility of directly converting sunlight into green hydrogen. This makes the solar energy storable and transportable in the form of hydrogen, an elegant energy source which provides electricity and heat efficiently, with water as the final emission [7]. The green hydrogen can be supplied as an energy source to the water sector—for water abstraction, conveyance, treatment and distribution—and to other sectors as well. The hydrogen generation and usage can be incorporated into the existing water infrastructures in either a centralized (power plant) or decentralized (district level or customer homes) manner. Examples of decentralized application are the green hydrogen filling stations set up at the solar panel fields next to KWR Water Research Institute, Nieuwegein, the Netherlands. As calculated, an 8.7 MWp solar park combined with rainwater collection and solar panels on roofs could supply the heat and half water demand for 900 houses and hydrogen for 540 hydrogen electric vehicles over the year, which would lead to a 3600-ton CO_2_eq/year reduction.

Urban water is a medium that carries thermal energy as it flows through modern cities: drinking water carries cold and wastewater carries heat. In Beijing, for example, 28 000 km of pipe distributes 4 billion m^3^ of drinking water across the city every year. If 1°C were to be recovered from the drinking water, it would represent a cooling-energy saving equivalent to ∼10% of the city's annual electricity production. In Amsterdam, the water cycle utility (Waternet) showed that by using surface water for office cooling and drinking water for blood bank cooling, cold recovery reduced GHG emission by 90%, while cost was decreased by 17% without sacrificing water quality [8]. Most of the energy consumption in the water sector involves the heating of water at the end-users’ premises [3]. If the water is first flowing through areas where cooling is required, it will take up heat. Subsequently, when it is used for e.g. washing, less heat is needed to heat it to washing temperature. Placing thermal energy ‘donors’ close to ‘users’ in future urban planning will make it possible to fully integrate the thermal energy-carrying capacity of water into current water distribution infrastructures. Moreover, using the water distribution and wastewater collection system to transport heat out of cities, instead of through the air by traditional air-conditioners, is not only more energy efficient than air-based cooling, but also increases wastewater temperature and helps optimize the commonly used biological treatment performance when reducing the urban heat island effects.

Wastewater processing is one of the main contributors to GHG emissions in the water sector (∼737 Mt CO_2_eq, 1.5% of total GHGs) [2]. With a view to achieving a circular economy, the new generation of wastewater treatment processes incorporates the recovery of resources, such as biogas for electricity and heat generation, and nutrients for reuse as fertilizer. In New England, most wastewater utilities had considerable success reducing their carbon footprint with anaerobic membrane bio-reactor (MBR) followed by anaerobic digestion and use of microturbines for electricity production or internal-combustion engines for combined heat and power (CHP). Estimated by US Energy Information Administration (EIA), 65 such wastewater treatment facilities produced ∼1 billion kWh of electricity in 2019. The combustion of biogas (mainly methane) produces CO_2_, while fertilizer reuse ends as agriculture emission of CO_2_, CH_4_ and N_2_O. Capturing C, N, P and S from wastewater to produce single-cell protein (SCP) as a human and animal food source would avoid such emission, and can be done by methane-oxidizing bacteria, hydrogen-oxidizing bacteria or purple non-sulfur bacteria [9]. In this regard, in China, iCell (Tiantai) has treated 2 Mm^3^ of brewery wastewater and produced thousands of tons of SCP product to be used, for instance, in pig farming. SCP production could also be driven from municipal wastewater. Moreover, the generated CH_4_ from the wastewater process could be further converted into graphite and hydrogen, which effectively captures carbon as a solid product, potentially contributing to a hydrogen-based water sector [10]. The treated wastewater can be reused when needed for drinking water, eventually by groundwater recharge, or for feeding water bodies to combat the heat island effects in urban areas.

The evidence to date from various parts of the water sector suggests that, far from being unavoidably energy intensive, the water sector can contribute significantly to GHG emission reduction. An essential condition for the transformation of current water systems into a ‘Blue Route’ for combating climate change is the integration of water, energy and resources, which could be implemented at community, district or city scales including one or all the elements. Although the individual technologies have been demonstrated case-by-case worldwide, the vital top-level design, systematic integration and innovative (big) data-based decision-making paradigm are still missing. Therefore, water scientists, policy makers and urban designers must work together to overcome the technical and non-technical barriers for putting the ‘Blue Route’ into practice, and to turn the water sector carbon neutral or even carbon negative.
